# The Re-Localization of Proteins to or Away from Membranes as an Effective Strategy for Regulating Stress Tolerance in Plants

**DOI:** 10.3390/membranes12121261

**Published:** 2022-12-13

**Authors:** Yee-Shan Ku, Sau-Shan Cheng, Ming-Yan Cheung, Cheuk-Hin Law, Hon-Ming Lam

**Affiliations:** School of Life Sciences and Centre for Soybean Research of the State Key Laboratory of Agrobiotechnology, The Chinese University of Hong Kong, Hong Kong SAR, China

**Keywords:** protein re-localization, protein lipidation, co-translational modification, post-translational modification, plant stress adaptability, transcriptional regulation, myristoylation, palmitoylation, prenylation

## Abstract

The membranes of plant cells are dynamic structures composed of phospholipids and proteins. Proteins harboring phospholipid-binding domains or lipid ligands can localize to membranes. Stress perception can alter the subcellular localization of these proteins dynamically, causing them to either associate with or detach from membranes. The mechanisms behind the re-localization involve changes in the lipidation state of the proteins and interactions with membrane-associated biomolecules. The functional significance of such re-localization includes the regulation of molecular transport, cell integrity, protein folding, signaling, and gene expression. In this review, proteins that re-localize to or away from membranes upon abiotic and biotic stresses will be discussed in terms of the mechanisms involved and the functional significance of their re-localization. Knowledge of the re-localization mechanisms will facilitate research on increasing plant stress adaptability, while the study on re-localization of proteins upon stresses will further our understanding of stress adaptation strategies in plants.

## 1. Introduction

In the natural environment, plants are constantly facing various abiotic and biotic stresses. The cell wall is the front line for stress perception. For example, mechanical stresses caused by injury or insect bites lead to the deformation of the cell wall, which then alters the contact between the cell wall and the cell membrane [1]. Hyperosmotic stress can cause plasmolysis, which is reversible in living plant cells. In addition to the cell membrane, within the cell, organelles are also surrounded by membranes. The dynamic properties of membrane proteins allow the sensing and relay of signals upon stresses.

Microscopic technologies and protein fractionation techniques enable the tracing and detection of protein re-localization upon stresses. Alterations in subcellular localization could be achieved by mechanisms including the cleavage of the signal peptide, interactions with membrane-localized proteins, and the lipidation of proteins. Some proteins possess the potential to bind to membranes due to features such as the pleckstrin homology (PH) domain and the protein kinase C conserved region 2 (C2) domain.

Common lipidation modifications of proteins include myristoylation, prenylation, palmitoylation, oleation, and glycosylphosphatidylinositol (GPI) anchoring. Among these mechanisms, myristoylation is the major protein lipidation mechanism in eukaryotes [2]. It is an irreversible co-translational or post-translational modification of proteins [2]. Myristoylation refers to the attachment of a myristoyl group to the N-terminal glycine of the protein through the formation of an amide bond mediated by N-myristoyltransferase (NMT) [3,4]. This modification is especially common in plants [5,6]. Prenylation refers to the attachment of a lipophilic farnesyl or geranylgeranyl group to a cysteine residue near the C-terminus of the protein [7]. Three types of prenyltransferases have been identified. Farnesyltransferase (FT) and geranylgeranyltransferase (GT) mediate the attachment of the farnesyl group and the geranylgeranyl group, respectively [8]. In addition, the attachment of two geranylgeranyl groups to the C-terminus of Rab proteins is mediated by Rab geranylgeranyltransferase (RGT) [9]. This modification allows the attachment of hydrophilic proteins to the hydrophobic membrane [7]. Palmitoylation refers to the attachment of fatty acids, usually palmitic acid, to the cysteine, serine, or threonine residues of a protein. The major type of palmitoylation is S-palmitoylation, which is the attachment of fatty acids to the cysteine residue [10]. Palmitoylation regulates the association of proteins with the plasma membrane, which is important for intracellular signaling [11]. For example, receptor-like kinases (RLKs) and heterotrimeric G proteins are major types of palmitoylated proteins [10]. The modification is catalyzed by protein S-acyltransferases (PATs), in which the DHHC-CRD domain is critical for the action of PATs [10,12,13]. Oleate is one of the most common monounsaturated fatty acids in plant cells, composed of 18 carbons with a cis double bond in the c-9 position [14]. The biosynthesis of oleate begins with acetyl coenzyme A (acetyl-CoA), which is converted to a saturated C18 product. After that, an acyl-carrier protein is conjugated to the C18 product by fatty acid desaturase to form the double bond [15]. Glycosylphosphatidylinositol (GPI) is a phosphoglyceride linked to the C-terminus of a protein. The structure of GPI mainly consists of three parts, including three mannoses, a glucosamine, and a phosphatidylinositol. GPI serves as a bridge between the protein and the lipid molecule [16,17,18,19]. The modification allows the attachment of proteins to lipid molecules, which then enables the protein to be anchored to membranes even if the protein does not contain any transmembrane domains. GPI anchoring is a post-translational modification that happens in the endoplasmic reticulum (ER) [16,18,20]. Various enzymes are involved in anchor synthesis and protein substrate modification [21].

Some proteins harbor functional domains for phospholipid binding, which is regulated by several factors, including the cellular level of calcium, the abundance of specific phospholipid species, and the local curvature of the membrane [22]. The two major groups of phospholipid-binding domains in proteins are the PH and protein C2 domains [22]. PH domain-containing proteins share low sequence homology. However, structurally, the proteins exhibit similar small modular structures including two perpendicular anti-parallel β-sheets followed by a C-terminal amphipathic helix. The PH domain binds to the phosphatidylinositols of biological membranes so that it helps to recruit proteins to different cellular compartments with specific phosphatidylinositols in their membrane composition under different cellular conditions [23]. The C2 domain comprises about 116 amino acid residues and is so named because of its location between the two copies of the C1 domain in protein kinase C and the catalytic domain. It is shown to have an affinity for a wide range of lipid components of cell membranes, including phosphatidylserine and phosphatidylcholine. 

The functional significance of the protein re-localization includes the regulation of the signal relay, gene expression, water transport, and the promotion of cell integrity (Table 1). The understanding of protein re-localization upon stresses will facilitate the delineation of stress-coping strategies in plants at the cellular level.

## 2. Protein Re-Localization upon Water-Related Stress

### 2.1. Enhanced Localization of Annexin 1 at the Cell Membrane upon Plasmolysis

Plasmolysis is an immediate cellular response under hypertonic conditions. In Arabidopsis, upon NaCl- or mannitol-induced plasmolysis of root epidermal cells, annexin 1-green fluorescent protein (ANN1-GFP) was found to be enriched at the plasma membrane and remained at the plasma membrane even after de-plasmolysis [24]. Annexins have been reported as positive regulators of abiotic and biotic stresses in plants [25]. It was suggested that the drop in cellular pH upon osmotic stress altered the hydrophobicity of annexins and their relocation, and the subsequent formation of oligomeric ion channels in the membrane [24]. The enhanced curvature of the plasma membrane upon plasmolysis was also hypothesized to be related to the clustering of ANN1-GFP and its interaction with the membrane [24]. It was suggested that the ANN1-GFP accumulation at the plasma membrane upon osmotic stress could promote the association with Hechtian strands and the reticulum, which could then encourage the attachment of protoplasts to the cell wall [26,27]. However, it is not clear whether the drop in cellular pH due to other stresses can also result in the plasma membrane localization of annexins.

### 2.2. De-S-Palmitoylation of MfNACsa to Activate Its Transcriptional Regulatory Function

NAC [NAM (no apical meristem), ATAF (Arabidopsis transcription activator factor) 1/2, and CUC (cup-shaped cotyledon) 2] transcription factors are known as major regulators of drought and salinity responses in plants [28]. *MfNACsa* was identified from a drought- and cold-tolerant diploid variety of *Medicago falcata*. The overexpression of *MfNACsa* in *Medicago truncatula* promoted drought tolerance [29]. In the unstressed condition, *MfNACsa* is *S*-palmitoylated and localized at the plasma membrane [29]. Under drought stress, *MfNACsa* is induced, with *MfNACsa* being de-*S*-palmitoylated and localized in the nucleus, where it activates the expression of *MtGly1* [29]. MtGly1 in turn promotes drought tolerance by maintaining the glutathione pool in a reduced state [29].

### 2.3. Redistribution of Aquaporins under Water-Related Stresses

Aquaporins are transmembrane proteins that facilitate the intercellular or intracellular movement of water and small neutral solutes [30,31]. Aquaporin isoforms include plasma membrane intrinsic proteins (PIPs), tonoplast intrinsic proteins (TIPs), nodulin 26-like intrinsic proteins (NIPs), small basic intrinsic proteins (SIPs), and uncharacterized intrinsic proteins (XIPs) [30]. Besides rice and Arabidopsis, which are the model plants for monocots and dicots, respectively, aquaporins are widely studied in poplar, which is a tree model for studying hydraulics [32]. Re-localizations of rice, Arabidopsis, and poplar aquaporins upon stress have been reported. In rice, the redistribution of OsPIP1;1, OsPIP2;4, and OsPIP2;5 away from the plasma membrane was observed in exodermal and mesodermal cells under salt stress or PEG-induced osmotic stress [33]. In Arabidopsis, upon salt stress, TIP1;1 was found to re-locate from the tonoplast to intracellular spherical structures [34]. However, the re-localization of poplar aquaporins to or away from membranes upon water-related stresses is relatively unclear. Instead, upon hypotonic stress, PtoPIP1;1 from poplar was found to exhibit polar-like localization at the plasma membrane compared to the relatively even distribution at the plasma membrane under normal conditions [35]. In addition, the ice plant has been employed as a model for studying abiotic stress in plants [36]. Upon osmotic stress, McTIP1;2 from *Mesembryanthemum crystallinum* was found to have promoted localization in the tonoplast [37]. The re-localization of aquaporins facilitates the regulation of water transport to cope with water-related stresses. However, the mechanism of the aforementioned redistribution is largely unknown. Nevertheless, in poplar, under excessive Zn, AQUA1 was found to be re-localized in new-forming pro-vacuoles while localizing on different membranes destined to form aggregates related to autophagic multivesicular bodies [38]. The post-translational phosphorylation of AQUA1 was suggested as the possible mechanism behind the re-localization, as the re-localization was disturbed by phosphatases and kinase inhibitors [38]. Although the re-localization of AQUA1 was not reported to be associated with water-related stresses, such a mechanism may set a reference for studying the re-localization of aquaporins under other stresses.

## 3. Protein Re-Localization upon Salt Stress

### 3.1. Reduction of Aquaporins in the Plasma Membrane upon Salt Stress

Salt treatment has been demonstrated to induce plasma membrane internalization [39,40]. This suggests that the trafficking of membrane proteins could be induced by salt stress. Using GFP as the reporter, the exocytosis and endocytosis of AtPIP1;2 and AtPIP2;1 were shown to be induced by salt stress, though the hypothesis of salt-induced massive internalization of PIPs was not supported [40]. However, in other studies in *Arabidopsis thaliana*, sorbitol-induced osmotic stress, NaCl-induced salt stress, and salicylic acid (SA) treatment were shown to reduce PIP accumulation in the plasma membrane [41,42]. Using GFP as the reporter, the movement of GFP-PIP2;1 during salt treatment was studied. Under salt stress, the Brownian diffusion, directed diffusion, and mixed trajectory of PIP2;1 were decreased compared to the unstressed condition, while restricted diffusion was increased [41]. Under normal conditions, the internalization of GFP-PIP2;1 was found to be dependent on endocytic pathways, including the clathrin pathway and, to a lesser extent, the membrane raft-associated pathway [41]. A higher density of GFP-PIP2;1 on the plasma membrane was detected upon the interruption of the endocytic pathway by TyrA23 (tyrphostin A23, a clathrin-mediated endocytic pathway inhibitor [43]), but not by other endocytic pathway inhibitors such as MβCD (methyl-β-cyclodextrin: a sterol inhibitor), Fen (fenpropimorph: a sterol synthesis inhibitor), and PPMP (_DL_-threo-1-phenyl-2-palmitoylamino-3-morpholino-1-propanol: a sphingolipid biosynthesis inhibitor) [41]. Upon salt treatment, the correlation between the GFP-PIP2;1 density and the endocytic pathways was enhanced [41]. When the endocytic pathway was interrupted by TyrA23 together with salt treatment, compared to salt treatment alone without TyrA23, the density of GFP-PIP2 on the plasma membrane was significantly increased [41]. A similar phenomenon was observed when the membrane raft-associated endocytic pathway was interrupted by MβCD, Fen, or PPMP [41]. The internalization of PIP proteins is likely related to the prevention of water loss from cells [44]. In addition, upon salt stress, using GFP as the reporter, the re-localization of TIP1;1 from the tonoplast into intracellular spherical structures was observed in Arabidopsis [34]. Interestingly, in the same study, the subcellular localization of TIP2;1 was found to be unchanged but remained associated with the tonoplast upon salt treatment [34]. It therefore appears that the re-localizations of proteins, even those with similar functions, are regulated by different mechanisms.

### 3.2. Recruitment of SOS2 to the Plasma Membrane upon Salt Stress

The SOS (salt overly sensitive) signaling cascade has been widely known to regulate salt tolerance [45]. SOS1, SOS2, and SOS3, a plasma membrane Na^+^/H^+^ exchanger [46], a protein kinase [47,48], and a calcium-binding protein [48], respectively, are the major components of the SOS signaling cascade. SOS3 could be detected in both soluble and membrane protein fractions [49]. Although N-myristoylation is essential for the function of SOS3, it does not dictate the membrane-association property of SOS3 [49]. SOS3 activates the protein kinase activity of SOS2 in a Ca^2+^-dependent manner and recruits SOS2 to the plasma membrane [48,50]. *SOS3* is only expressed in the root but not the shoot, while *SOS2* is expressed in both roots and shoots [51]. Such expression patterns imply that SOS2 may be regulated by other proteins in tissues in which *SOS3* is not expressed. Using yeast as the model, it was found that SCABP8 (SOS3-LIKE CALCIUM BINDING PROTEIN8) can also recruit SOS2 to the plasma membrane and activate SOS1 [51]. Later, it was found that, upon salt stress, VPS23A (vacuolar protein sorting 23A) positively regulates the plasma membrane localization of SOS2 [52]. Without stress, SOS2 is distributed throughout the whole cell [52]. Upon salt stress, SOS2 has enhanced localization on the plasma membrane [52]. However, when *VPS23A* was mutated, SOS2 did not show enhanced plasma membrane localization upon salt stress [52]. The mutation of *VPS23A* also led to the increased salt sensitivity of Arabidopsis plants [52]. However, in the *vps23A* mutant, when SOS2 was artificially designed to localize to the plasma membrane by overexpressing *SOS2* with the myristoylation sequence, the transgenic plants had better tolerance to salt compared to the *vps23A* mutant background [52]. The results suggest the importance of the plasma membrane localization of SOS2 in conferring salt tolerance [52]. The phenomenon is consistent with the notion that SOS2 activates SOS1, a membrane-bound Na^+^/H^+^ exchanger. Although the SOS2 protein does not have a canonical membrane localization signal, the regulated localization of SOS2 upon stress enables the functional plasticity of SOS2, which is a protein kinase that can activate other proteins [52].

### 3.3. Stabilization of MdCBL1 at the Plasma Membrane by Palmitoylation upon Salt Stress

In apples, upon salt stress, the expression of *MdPAT16* (*Malus domestica palmitoyltransferase 16*) is induced and is a positive regulator of salt tolerance and sugar accumulation [53]. MdPAT16 palmitoylates MdCBL1 (*Malus domestica* calcineurin B-like 1) to mediate the plasma membrane localization of MdCBL1, which is a positive regulator of sugar accumulation [53]. MdCBL1^C3S^, which had a mutated palmitoylation site, was found to be mislocalized to the cytoplasm and nucleus [53]. It was therefore concluded that MdPAT16 stabilizes the plasma membrane localization of MdCBL1 by palmitoylation upon salt stress [53].

## 4. Protein Re-Localization upon Heat/Cold Stress

### 4.1. Promotion of DnaJ Lipidation by Heat Shock

Heat stress induces endoplasmic reticulum (ER) stress, resulting from the aggregation of misfolded proteins [54], which are bound and stabilized by heat shock proteins (HSPs). The heat stress factor (HSF) is localized in the nucleus and regulates gene expression by recognizing heat stress promoter elements [55,56]. Protein modifications, especially those on HSPs, are found to be important for heat stress tolerance [57]. DnaJ, which is also known as HSP40, is localized to the membrane by farnesylation and geranylgeranylation [58,59]. Heat shock promotes the prenylation of DnaJ and thus the enrichment of DnaJ proteins at the membrane [58,59]. The enrichment of prenylated DnaJ was suggested to be positively correlated to heat tolerance [58,59].

In Arabidopsis, AtJ3 is identified as a cytosolic HSP40 family member that contains a CaaX box for farnesylation and mediates the protein farnesylation-dependent response to heat stress [60]. AtJ3 farnesylation facilitates its association with the membrane [61]. Arabidopsis *j3* mutants with abolished CaaX boxes failed to produce farnesylated AtJ3. Compared to the wild type, the *j3* mutant exhibited intolerance to prolonged exposure at 37 °C for 4 days but improved tolerance to sudden heat shock at 44 °C for 30 min. Such responses to heat stress are similar to those of the Arabidopsis *hit5/era1* (*heat-intolerant 5/enhanced response to aba*) mutant that has a mutated β-subunit of the protein farnesyltransferase (PFT), which mediates protein farnesylation [60,62]. Moreover, the mutant form of protein farnesyltransferase encoded by *hit5/era1* resulted in an abscisic acid (ABA)-independent but temperature-dependent phenotype [63]. Previous findings have suggested that J-proteins improve thermotolerance in Arabidopsis by functioning as a component of HSP70/HSP40-based chaperones under heat stress [62,64]. Compared to the wild type, the *j3* mutant had an increased accumulation of insoluble proteins in cells when subjected to heat stress [62]. It has been reported that AtJ3 and HSP70-4 co-operate to mediate heat stress tolerance in Arabidopsis [62]. A subcellular localization study showed that EYFP^N^-HSP70-4 and EYFP^C^-J3 interacted to reconstitute EYFP, which was condensed in the membrane-less heat stress granules under heat stress while showing diffused localization in the cell under physiological temperature [62]. Although the interaction between AtJ3 and HSP70 was farnesylation-independent, it was proposed that the farnesylated site of AtJ3 is required for directing HSP70 to the hydrophobic residues on the heat-denatured proteins, and assisting in protein refolding for heat stress alleviation [62].

Other than assisting in protein refolding in conjunction with HSP70-4, AtJ3 is also a component of the membrane-bound RISC (RNA-induced silencing complex) in Arabidopsis. AtJ3 farnesylation promotes membrane association by interacting with AGO1, an effector protein in microRNA (miRNA)- and small interfering RNA (siRNA)-mediated gene silencing in Arabidopsis [65]. It was reported that *era1* or the *AtJ2/AtJ3* mutant had increased levels of the miRNA-associated membrane-bound polysomes with impaired noncell autonomously acting siRNA gene silencing, suggesting the potential function of the post-translational modification of AtJ3 in regulating translation and leading to the differential stress tolerance among different Arabidopsis genotypes [65].

### 4.2. Protein Re-Localization by Transmembrane Domain Cleavage to Regulate Gene Expressions upon Heat Stress

Under heat stress, the membrane-tethered bZIP28 is re-localized to the nucleoplasm as a result of the heat-induced cleavage of the C-terminus by site 1 or site 2 proteases to remove its transmembrane domain [66]. In the nucleoplasm, the re-localized bZIP28 up-regulates heat stress-coping genes such as the one encoding the ER-localized chaperone, BiP2, in Arabidopsis [66,67]. A similar proteolytic activation of transcription factors under heat stress was also reported in rice. A rice NAC transcription factor, OsNTL3, carries a predicted C-terminal transmembrane domain [68]. Under heat stress and ER stress, the membrane-localized OsNTL3 is re-localized to the nucleus and binds to the promoter region of *OsbZIP74* for stress response activation [68]. 

### 4.3. Alteration of Protein Localization by mRNA cleavage upon Heat Stress

The inositol-requiring enzyme 1 (IRE1)-mediated bZIP mRNA splicing for the proteolytic activation of bZIP transcription factors is conserved in plants [69,70]. In Arabidopsis, the ER-localized inositol-requiring enzyme 1 (AtIRE1) is responsible for the unconventional mRNA splicing of *AtbZIP60* under heat stress, which leads to a shift in the open reading frame that promotes the nuclear localization of the protein instead of localization at the membrane [69]. Nuclear localization enables the transcriptional regulatory function of AtZIP60 to modulate gene expression [69]. In rice, the mRNA of *OsbZIP74* was also spliced by OsIRE1 when under heat and ER stress [70]. The cleavage of the C-terminal transmembrane domain from the transcript of *OsbZIP74* promotes nuclear localization and thus enables the transcriptional regulatory function of the protein [70].

### 4.4. Translocation of Proteins to the Nucleus to Regulate Gene Expressions upon Cold Stress

In Arabidopsis, a cytosolic redox protein, thioredoxin h2 (Trx-h2), was reported to be involved in redox-mediated regulation and structural switching upon exposure to cold stress [71]. Under normal conditions, Trx-h2 is anchored to the cytoplasmic endomembrane via the myristoyl group that is covalently attached to Gly2. However, under cold exposure, the de-myristoylation of Trx-h2 facilitates nuclear localization by exposing the nuclear localization signal (NLS) located at the C-terminus [71]. Trx-h2 reduces the disulfide bonds of the inactive CBF oligomers to release the active monomers, which then bind the promoter regions of cold-responsive (COR) genes to activate their expression [71,72]. Therefore, the translocation of Trx-h2 into the nucleus upon exposure to cold stress favors the expression of cold-related genes and promotes cold stress tolerance [71]. The myristoylated plasma membrane-localized Clade-E growth-regulating 2 (EGR2) phosphatase is also involved in regulating the expression of cold stress-coping genes in Arabidopsis [73]. EGR2 interacts with Open Stomata 1 (OST1), which is a positive regulator of *CBF* gene expressions and inhibits the activity of OST1 under normal conditions [73]. At 22 °C, EGR2 was found to be N-myristoylated by N-myristoyl-transferase 1 (NMT1) and detected in the plasma membrane but not in the soluble fraction [73]. When EGR2 was mutated to inhibit N-myristoylation, the mutated egr2 lost the membrane-binding specificity and was detected in the plasma membrane, cytosol, and nucleus at 22 °C [73]. At 4 °C, EGR2 was detected in all plasma membranes, the cytosol, and the nucleus [73]. It was therefore concluded that the plasma membrane localization of EGR2 was dependent on N-myristoylation, which was hampered at 4 °C. The N-myristoylation of EGR2 is important for its interaction with OST1. Upon binding to OST1, the N-myristoylated EGR2 dephosphorylates OST1 to repress its kinase activity, which is important for the activation of CBF pathways to achieve freezing tolerance. Under cold stress, the interaction between EGR2 and NMT1 is weakened, and the N-myristoylation of EGR2 by NMT1 is suppressed. Without being bound by N-myristoylated EGR2, the kinase activity of OST1 is not repressed. Consequently, the CBF pathway is activated to achieve freezing tolerance [73].

The Salt Tolerance Related Protein (STRP) could be found in the cytosol, nucleus, or plasma membrane [74]. However, under cold stress, the amount of STRP in the membrane fraction decreases while it increases in the cytosol and nucleus [74]. The association of the STRP with membranes is proposed to be regulated by lipid attachment or by anchoring to plasma membrane-localized proteins [74]. As the *strp* mutant is more susceptible to oxidative damage with increased lipid peroxidation and altered membrane integrity compared to the wild type, it is proposed that the increased localization to the nucleus might help modulate the expression of the specific cold-activated gene or represent a protection mechanism in response to reactive oxygen species (ROS) production upon cold stress [74].

### 4.5. Changes in Protein Subcellular Localization in Response to Heat/Cold Stress-Induced Ca^2+^ Signaling

In response to the sudden change in temperature, Ca^2+^-permeable channels mediate signals that lead to an influx of Ca^2+^ into plant cells [75,76,77]. By regulating the calcium signaling, the tolerance of the plant to temperature fluctuations could be improved [78,79]. An important function of calcium-dependent protein kinase (CDPK or CDK) is to perceive changes in the cytosolic calcium concentration in response to external stimuli [80]. The expression level of the membrane-localized calcium-dependent protein kinase is often correlated to the responses to heat, cold, and wounding stress in the plant [81,82,83,84,85,86,87]. The activation of the downstream cold-regulated (COR) gene expressions for better cold stress adaptation requires the cytosolic Ca^2+^ signal [88]. The membrane-localized ZmCDPK7 is a heat-response kinase in maize that participates in ABA signaling and heat stress tolerance by phosphorylating sHSP17.4 [89]. ZmCDPK7 contains N-terminal myristoylation and palmitoylation sites with no transmembrane region for anchoring to the membrane. It is reported that there is a shift in the membrane-localized ZmCDPK7 to the cytoplasm under heat-stress conditions [90]. It is proposed that the ZmCDPK7 activates the sHSP17.4 via phosphorylation in the cytoplasm for maintaining protein stability, while the sHSP17.4 was previously reported to be heavily phosphorylated under heat and drought conditions in maize [89,91].

## 5. Protein Re-Localization upon Mechanical Stress for Protein Activation

Several calcium-dependent protein kinases are systemically induced upon wounding, suggesting the possible involvement of these proteins in wounding and herbivory responses. From a genome-wide analysis of calcium-dependent protein kinases in *Glycine max*, two genes encoding membrane-localized CDPKs, *GmCPK3* and *GmCPK31*, showed enhanced expression under wounding and herbivory stresses [92]. In Arabidopsis, a herbivory-induced phytohormone-independent pathway mediated by the CPK cascade was associated with better defense against wounding and herbivory [93]. AtCPK3 and AtCPK13 activate a member of the heat stress transcription factor family, HsfB2a, via phosphorylation, which then activates the expression of herbivory-inducible defense-related genes [93]. In tomatoes, the expression of *LeCDPK1* was rapidly and transiently enhanced both locally at the site of the injury and systemically in the distant non-wounded leaves [94]. Similar to tomatoes, both the kinase activity and the mRNA of *ZmCPK11* increased as a systemic response to wounding in maize [95]. Unlike other CPKs such as AtCPK1 and DcCPK1 (*Daucus carota* CPK1), ZmCPK11 lacks a myristoylation/palmitoylation site [95]. However, it was found that ZmCPK11 was activated via interactions with phospholipids [95]. After injury, the activity of the membrane-bound CDPK relative to the total CDPK activity was increased two-fold [94,96]. The translocation of ZmCPK11 from the cytosol to membranes and the increased activity of ZmCPK11 upon its binding to phospholipids were proposed to be the possible mechanisms behind the wounding response in maize [95].

In rice, a GTPase-activating protein 1 (OsGAP1) was reported to activate the GTPase activity of OsYchF1 [97]. Under mechanical stress and in the presence of Ca^2+^, OsYchF1 was localized to the plasma membrane via its interaction with OsGAP1 [98]. It is proposed that the re-localization of OsGAP1 is associated with the functional activation of OsYchF1 to promote resistance to wounding and pathogen challenges [97,98].

## 6. Protein Re-Localization upon Biotic Stress

Rice *OsERG1* contains a single C2 domain, which is responsible for calcium-dependent phospholipid binding. In one study, the transcript level of *OsERG1* was induced by the elicitors of a fungal blast, *Magnaporthe grisea*. In addition to inducing transcription, the elicitor treatment also led to the subcellular re-localization of OsERG1 from the cytosol to the plasma membrane, although the mechanism of the re-localization was unclear. By either elevating the subcellular calcium level or applying a calcium-mobilizing agonist (A23187), the plasma membrane localization of OsERG1 was either induced or suppressed, respectively [99]. In wheat, TaERG3 also harbors a C2 domain. The transcript level of *TaERG3* was inducible by ABA treatment, high salinity, cold treatment, an increased level of calcium, and infection by *Puccinia striiformis* f. sp. *tritici*, which causes stripe rust. *TaERG3* is characterized as a positive regulator of ABA signaling and salt and cold stress. *TaERG3* also enhances the resistance towards *Puccinia striiformis* f. sp. *tritici*. *TaERG3* is localized in the plasmalemma and nucleus [100]. However, the effect of stress on the subcellular localization of TaERG3 remains unclear.

Upon biotic stress, chloroplasts are the major sites for the production of antimicrobial reactive oxygen species (ROS) and the biosynthesis of defense hormones, including SA and jasmonic acid (JA) [101]. Plant cells usually perceive biotic threats at the cell surface. Upon pathogen perception, the chloroplast thylakoid membrane-associated calcium-sensing receptor (CAS) is stimulated and activates pattern-triggered immunity (PTI) [102]. CPKs are major components of calcium signaling [103]. In Arabidopsis, CPK16 localizes along the plasma membrane through N-myristoylation under normal conditions. The N-myristoylation site of CPK16 overlaps with the chloroplast transit peptide (cTP). Upon flagellin 22 (flg22) treatment, CPK16 re-localizes from the plasma membrane to the chloroplast to promote chloroplast-dependent defenses [101]. At the same time, pathogen effectors also re-localize from the plasma membrane of the infected plant cell to the chloroplast to suppress defense response including hormone biosynthesis [101]. In a study on the tomato—*tomato yellow leaf curl virus* (TYLCV) interactions, the C4 protein from TYLCV was found to have the N-myristoylation site overlap with the chloroplast transit peptide [101]. Upon the activation of the defense response triggered by the replication-associated viral protein (Rep), the bacterial elicitor peptide flg22, or the plant peptide Pep1, the TYLCV C4 protein is re-localized to the chloroplast from the plasma membrane [101]. Once it has entered the chloroplast, the TYLCV C4 protein interacts with and suppresses the function of the thylakoid membrane-associated CAS, which is involved in PTI-induced transcriptional reprogramming, SA biosynthesis, callose deposition, and anti-bacterial and anti-fungal resistance [101]. The simultaneous re-localization of both the protective protein from the plant and the pathogenic protein from the virus to the chloroplast shows the continuous battle between the plant and the virus.

## 7. Protein Re-Localization upon Oxidative Stress

Oxidation is a secondary stress resulting from both abiotic and biotic stresses [104]. Hydrogen peroxide (H_2_O_2_) is one of the most common reactive oxygen species (ROS) in plants [105]. The transmembrane domains, TM2 and TM3, of PIP2 dictate the plasma membrane localization of PIP2. Although PIP1s lack plasma membrane localization signaling and are unable to localize to the plasma membrane on their own, their interactions with PIP2 allow them to be localized to the plasma membrane [106]. In *A. thaliana*, H_2_O_2_ treatment on roots led to the reduced accumulation of AtPIP1 and AtPIP2 in the plasma membrane [42,107], but they were re-localized to intracellular structures tentatively identified as vesicles and small vacuoles instead [42]. In the root, catalase treatment could counteract the effect of SA or salt on the internalization of PIP2;1 from the plasma membrane, suggesting the involvement of H_2_O_2_ in PIP re-localization upon stress [42].

## 8. Protein Re-Localization in Response to Stress Hormones

### 8.1. Regulation of NMT1 Expression by ABA

ABA regulates numerous plant growth, development, and stress response mechanisms, including seed dormancy, leaf senescence, responses against pathogen infection, and osmotic, drought, oxidative, and salt stresses [108,109,110,111]. When the plant is under stress, the ABA level increases and is perceived by pyrabactin resistance 1 (PYR1)/PYR1-like (PYL)/regulatory components of ABA receptors (RCAR) proteins, leading to the degradation of the negative regulators of ABA, ABI1, and PP2CA, by ubiquitination with PUB12/13 and RGLG1/5 E3 ligase, respectively [112]. In normal conditions, RGLG1 is N-myristoylated and bound to the plasma membrane. Upon ABA or salt treatment, the expression of *NMT1*, which is responsible for the N-myristoylation of RGLG1, is down-regulated. As a result, without being myristoylated, RGLG1 is shuttled from the plasma membrane to the nucleus, where it mediates the ubiquitination of the nuclear-localized PP2CA [113].

### 8.2. Mediation of Brassinosteroid Signaling by Myristoylated BSK1

Brassinosteroid (BR) plays roles in diverse cellular biological processes, including cell division, the cell cycle, morphogenesis, reproduction, and various stress responses [114]. BR is thought to act as a switch between normal metabolic activities and stress responses [114]. This role of BR as a master switch relies on brassinosteroid signaling kinase 1 (BSK1), which associates with the receptor kinases, BR-insensitive 1 (BRI1) or flagellin-sensing 2 (FLS2), and thus functions in both BR-regulated plant growth and flg22-triggered immunity. Upon BR perception, BRI1 phosphorylates BSK1 in the membrane raft. The phosphorylated BSK1 in turn activates BRI1 suppressor 1 (BSU1) and dephosphorylates BR-insensitive 2 (BIN2) to trigger downstream signaling. When flg22 molecules from invading pathogens are recognized by FLS2, BSK1 is relocated to the non-membrane raft with mitogen-activated protein kinase kinase kinase (MAPKKK) to trigger defense responses [115]. Therefore, the lining of BSK1 along the cytosolic side of the plasma membrane is crucial for interacting with BRI1 or FLS2 and triggering its master key function. This plasma membrane association is achieved by N-myristoylation. The BSK1^G2A^ mutant proteins, with the loss of the N-myristoylation site, are mainly distributed in the cytoplasm and endoplasmic reticulum [116]. Moreover, the BSK1^G2A^ mutant protein is unstable and subject to degradation by autophagy [116].

## 9. Protein Re-Localization in Response to Other Signaling Events

### 9.1. Regulation of SnRK Signaling by N-Myristoylation

Sucrose nonfermenting 1-related kinase 1 (SnRK1) is a key signaling molecule regulating cellular metabolism under both stress and growth-promoting conditions [117]. It is a heterotrimeric complex comprising an α-catalytic, and β- and γ-regulatory subunits [117]. SnRK1 functions are induced by stress conditions (including nutrient starvation and biotic and abiotic stresses) and switch the cellular metabolism from anabolism to catabolic pathways such as autophagy or stress responses [117]. SnRK1 functions by directly phosphorylating cytosolic metabolic enzymes or by phosphorylating the transcription factors in the nucleus that regulate nuclear-encoded plastid and mitochondrial genes, which in turn alter cellular metabolic states. Transcription factor genes targeted by SnRK1 include *bZIP63*, *FUS3*, *IDD8*, *EIN3*, *WRI1*, and *MYC2*, and they are involved in diverse metabolic and hormonal signaling pathways, such as sugar signaling, amino acid starvation, seed maturation and germination, oil synthesis, flowering time, ethylene, and ABA and JA responses [117]. When phosphorylated by SnRK1, these transcriptional factors either lose their ability to regulate transcription or bind DNA or are even degraded. To phosphorylate the transcription factors, SnRK1 has to be localized in the nucleus. The cellular localization of SnRK1 relies on its N-myristoylation status [118]. In Arabidopsis, the SnRK1 β1 subunit is the target site for N-myristoylation by NMT1 [118]. Myristoylated SnRK1 is sequestered to the plasma membrane and is thus unable to phosphorylate its target transcription factors [118]. During plant development, the different levels of *NMT1* expression in different tissues exert tissue-specific regulation on the activity of SnRK1 to coordinate the proper overall development of the plant [118].

### 9.2. Light/Sugar Sensing

Calmodulin (CaM53) from *Petunia hybrida* Mitchell is found to be geranylated by geranylgeranyl transferases (GGTase-I) and is thus plasma membrane-bound. However, under dark or low sugar level conditions, CaM53 is not geranylated and localizes in the nucleus. This nuclear localization of CaM53 leads to stunted growth, decreased stem internode length, leaf curling, chlorosis, and necrosis [119]. However, the detailed role of CaM53 in signaling remains unclear [119].

## 10. Discussion and Conclusions

**Figure 1:**
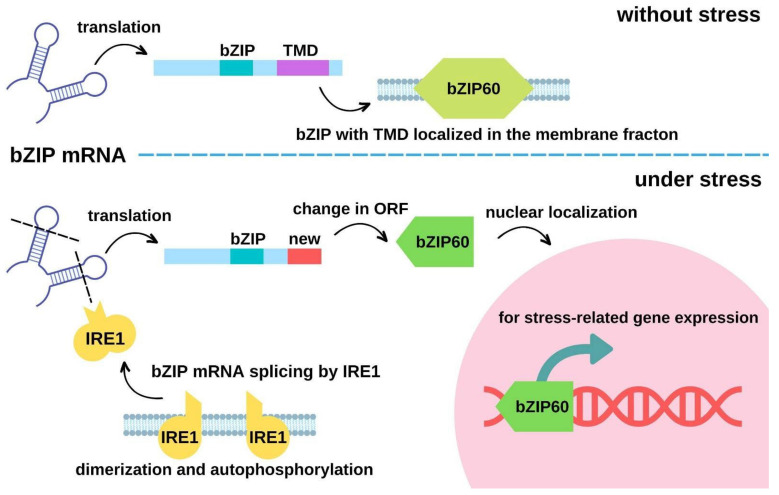
A diagram illustrating an example of the transcriptional regulations by re-localized proteins upon stress. TMD, transmembrane domain; ORF, open reading frame.

Various proteins re-localize to or away from membranes upon stresses. They include aquaporin water channels for regulating water transport, structural proteins for regulating cell integrity, chaperones for assisting in protein folding, signaling proteins for regulating Ca^2+^ and hormone signaling, and transcription-related proteins for regulating gene expression. An example of the transcriptional regulation resulting from protein re-localization is shown in Figure 1. The specific protein re-localizations and their functional significance are summarized in Table 1. Although myristoylation is irreversible, most of the other protein re-localization mechanisms, such as interacting with other biomolecules and lipidation, are reversible. Such reversibility enables the timely and plastic functional regulation of stress responses.

Upon re-localization, several proteins function by modulating up-stream cellular events. For example, in *A. thaliana*, the de-myristoylation of the SnRK1 β1 subunit facilitates the transcriptional regulation of genes including *bZIP63, FUS3, IDD8, EIN3, WRI1*, and *MYC2*. Among these targets, *FUS3* is annotated as a kinase for signal transduction, while the other genes are annotated as transcription factors. The re-localization of SnRK1 probably initiates a vast number of cellular responses. Other examples of the modulation of up-stream cellular events include the regulation of BR signaling by the re-localization of BSK1 upon biotic stress and the regulation of ABA signaling by the re-localization of RGLG1 upon ABA or salt treatment. Since BR and ABA are stress hormones that regulate various cellular events, the re-localization of BSK1 and RGLG1 probably results in the regulation of a vast number of cellular events. These examples show that the effects on cellular events from the re-localization of certain proteins can be greatly amplified in the downstream pathways. The modification of sub-cellular localization, therefore, appears to be an effective strategy for regulating the stress adaptability of plants. However, the mechanisms underlying the re-localization of a number of proteins upon stress have remained unknown. On the other hand, a number of proteins, such as ANJ, EGR2, HvFP1, and HIPP26, are lipidated upon stress (Table 1). However, the mechanisms for the re-localization of the proteins upon stress have remained unclear. Investigations of the re-localization mechanisms will facilitate the research on plant stress adaptability, while the study on the re-localization of proteins upon stress will further our understanding of stress adaptation strategies in plants.

Studies on the re-localization of proteins to or away from membranes are largely biased toward model crops such as Arabidopsis and rice. Moreover, many of the studies on the re-localization of proteins upon stresses are on aquaporins, which are involved in the transport of various molecules including water and neutral solutes. It is therefore reasonable that aquaporins are associated with the adaptation to various stresses including water-related stress and salt stress. Although poplar has been employed as the model for studies on aquaporins, the specific studies on the re-localization of poplar aquaporins upon stresses are less popular than those in Arabidopsis and rice. As mentioned above, although the functions of the proteins may appear to be similar, the re-localization upon stress could be different. It is therefore worthwhile to expand the studies on different proteins and different plant species. Scattered studies on the re-localization of proteins to or away from membranes were carried out in other plants, such as ice plants (*M. crystallinum*), apples (*M. domestica*), saltbushes (*A. nummularia*), maize (*Z. mays*), barley (*H. vulgare*), wheat (*T. aestivum*), and garden petunias (*P. hybrida*) (Table 1). Although these species are less common for studies on stresses compared to Arabidopsis, rice, and poplar, they are advantageous for studying specific mechanisms. For example, apples have been employed to study sugar accumulation in fruit while garden petunias have been employed to study flower color modification.

In conclusion, a vast number of proteins re-localize in the cell upon abiotic and biotic stresses. Mechanisms for such re-localization include the cleavage of the signaling peptide, interactions with membrane-localized proteins, and the lipidation of proteins. The functional significance of such re-localization includes regulating molecular transport, cell integrity, protein folding, signaling, and gene transcription. By regulating signaling and transcription, the effects brought about by protein re-localization can be greatly amplified. Thus, protein re-localization appears to be an effective strategy for promoting plant stress adaptability. The different re-localization mechanisms of protein homologs and the largely unexplored protein re-localization in non-model plants upon stress leave considerable room for future research.

**Table 1:** Summary of proteins that re-localize in plant cells upon stress.

Type of Stress	Species	Protein	Description of the Relocation	Mechanism of the Relocation	Functional Significance of the Relocation	References
Water- related stress	*Arabidopsis thaliana*	ANN1	Accumulation of ANN1 upon osmotic stress (plasmolysis)	Unknown	Association with Hechtian strands and reticulum at the plasma membrane for protection against osmotic stress	[24]
	*Medicago falcata*	MfNACsa	Relocated from the plasma membrane to the nucleus upon drought stress	De-*S*-palmitoylation of MfNACsa	MfNACsa activates the expression of *MtGly1* in the model plant *Medicago truncatula* for maintaining the glutathione pool in the reduced state to achieve drought tolerance	[29]
	*Oryza sativa*	OsPIP1;1, OsPIP2;4, and OsPIP2;5	Relocated away from the plasma membrane	Endocytosis of OsPIP2;5 is enhanced by salt stress	Regulation of water transport	[33]
	*Populus tomentosa*	PtoPIP1;1	Exhibited polar-like localization at the plasma membrane compared to the relatively even distribution at the plasma membrane under normal conditions	Not mentioned	Regulation of water transport	[35]
	*Mesembryanthemum crystallinum*	McTIP1;2	Exhibited promoted localization in the tonoplast	Not mentioned	Promotion of osmotic adjustment in the cell	[37]
Salt stress	*Arabidopsis thaliana*	SOS2	Enhanced plasma membrane localization upon salt stress	The plasma membrane localization is enhanced by VPS23A	For the activation of SOS1, a membrane-bound Na^+^/H^+^ exchanger	[52]
	*Malus domestica*	MdCBL1	Stabilized plasma membrane localization upon salt stress	The expression of *MdPAT16* is induced upon salt stress. MdPAT16 mediates the plasma membrane localization of MsCBL1 by palmitoylation	Regulation of sugar accumulation	[53]
	*Arabidopsis thaliana*	AtPIP2;1	Reduced plasma membrane localization upon osmotic stress and salt stress	Enhanced internalization of AtPIP2;1 upon water-deficit stress through endocytic pathways	Regulation of the water permeability of plasma membrane to protect cells from water-deficit stress	[41]
	*Arabidopsis thaliana*	TIP1;1	From the tonoplast to intracellular spherical structures	Not mentioned	Regulation of water transport inside the cell	[34]
Heat stress	*Arabidopsis thaliana*	AtJ3	From cytosol to membrane-less heat stress granules	Not mentioned	For the formation of HSP70/HSP40-based chaperones. Mutants failed to undergo AtJ3 farnesylation, leading to heat stress intolerance. AtJ3 farnesylation is responsible for directing HSP70 to the misfolded protein	[52,54,56]
	*Arabidopsis thaliana*	AGO1; AtJ2/AtJ3	From cytosol to membrane	Proposed model of farnesylation promotes the AtJ3-membrane interaction and AGO1-membrane interaction via J3, which further alters the loading of AGO1-miRNA to the polysome	Farnesyl transferase-deficient and farnesylation-deficient *j2/j3* mutants had increased levels of the miRNA-associated membrane-bound polysomes	[65]
	*Arabidopsis thaliana*	SKD1	From cytosol to messenger ribonucleoproteins	Not mentioned	Possibly involved in the selection of proteins to be localized to mRNP under stress conditions	[120]
	*Arabidopsis thaliana*	bZIP28	From membrane to endoplasmic reticulum and cytosol	Heat-induced cleavage at the membrane- tethering C-terminus	The re-localized bZIP28 up-regulates heat stress-coping genes such as the ER-localized chaperone BiP2 in Arabidopsis for coping with heat stress	[66,67]
	*Arabidopsis thaliana*	AtbZIP60	From membrane to nucleus	The ER-localized inositol-requiring enzyme 1 (AtIRE1) mediates the unconventional mRNA splicing of *AtbZIP60* by open reading frame shift	Membrane-localized active AtbZIP6 promotes the expression of stress-related genes	[69]
	*Atriplex nummularia*	ANJ1	Not mentioned	Not mentioned	Heat shock enhances the amount of the prenylated DnaJ protein in the membrane fraction; potentially functions in heat tolerance	[58]
	*Oryza sativa*	OsNTL3	From membrane to nucleus	Not mentioned	OsNTL3 binds to the promoter region of *OsbZIP74* for stress response activation	[68]
	*Oryza sativa*	OsbZIP74	From membrane to nucleus	The ER-localized inositol-requiring enzyme 1 (OsIRE1) cleaves off the C-terminal transmembrane domain of OsbZIP74	Membrane-localized active OsbZIP74 promotes the expression of stress-related genes	[70]
	*Zea mays*	ZmCDPK7	From membrane to cytosol	Not mentioned	ZmCDPK7 activates sHSP17.4 via phosphorylation in the cytoplasm for maintaining protein stability	[90,91]
Cold stress	*Arabidopsis thaliana*	Trx-h2	From membrane to nucleus	De-myristoylation of Trx-h2	Trx-h2 reduces the disulfide-bonded inactive CBF oligomers to form the active monomers that bind the promoter regions of cold-responsive (COR) genes	[71,72]
	*Arabidopsis thaliana*	EGR2	Not mentioned	Not mentioned	Low temperature attenuates the formation of the NMT1-EGR2 protein complex, leading to the suppression of the myristoylation of EGR2, and releasing its inhibition on OST1 for the proper activation of the CBF pathway and freezing tolerance	[73]
	*Arabidopsis thaliana*	STRP	Decrease in the membrane fraction of STRP	Not mentioned	Potentially affects the expressions of cold-activated genes, protects the chromatin structure, and stabilizes the membrane structure	[74]
	*Hordeum vulgare*	HvFP1	Not mentioned	Not mentioned	Farnesylation of HvFP1 is important for its precise nuclear localization	[121]
	*Arabidopsis thaliana*	HIPP26	Not mentioned	Not mentioned	Isoprenylation of HIPP26 is important for its precise nuclear localization	[122]
Mechanical stress	*Oryza sativa*	OsYchF1	From cytosol to membrane	Interact with the membrane-anchored interacting partner, OsGAP1	Proposed re-localization of the negative regulator of stress to alleviate stress susceptibility	[97,98]
Biotic stress	*Arabidopsis thaliana*	CPK16	From plasma membrane to chloroplast	Removal of N-myristoylation of RGLG1	Allows CPK16 to work in chloroplast and enhances the resistance towards *Pseudomonas syringae* pv. *tomato* DC3000 and *tomato yellow leaf curl virus*	[101]
	*Oryza sativa*	OsERG1	From cytosol to plasma membrane	Elevation of cellular calcium level	*OsERG1* is induced by elicitor from the fungal blast *Magnaporthe grisea* and is believed to play roles in fungal disease defense responses. The OsERG1 protein is relocated from cytosol to plasma membrane upon fungal elicitor treatment and calcium signals	[99]
	*Arabidopsis thaliana*	BSK1	From plasma membrane to cytoplasm and endoplasmic reticulum	Loss of N-myristoylation	BSK1 is plasma membrane-bound and interacts with BRI1 and FLS2 for triggering BR signaling or defense response. When flagellin is perceived, BSK1 would relocate to the non-membrane raft for functioning. If BSK1 fails to be modified by N-myristoylation, it would no longer associate with the plasma membrane and would be degraded through the autophagic pathway	[115,116]
Biotic stress and hormone	*Triticum aestivum*	TaERG3	From nucleus to plasma membrane	Increase in cellular calcium level	TaERG3 plays roles in ABA signaling and acts as a positive regulator of responses to high salt and low temperature. It also enhances resistance towards *Puccinia striiformis* f. sp. *tritici* (the pathogen causing stripe rust). It is predominantly localized in plasmalemma and nucleus	[100]
Stress hormone	*Arabidopsis thaliana*	RGLG1	From plasma membrane to nucleus	Reduction of N-myristoylation of RGLG1	Allows the binding of RGLG1 to PP2CA in the nucleus which is a negative regulator of ABA signaling	[113]
Nutritional starvation, biotic and abiotic stresses	*Arabidopsis thaliana*	SnRK1	From plasma membrane to nucleus	Removal of N-myristoylation of SnRK1 β1 subunit	Allows the binding of SnRK1 to its transcription factor targets including bZIP63, FUS3, IDD8, EIN3, WRI1, MYC2 in the nucleus. Upon phosphorylation by SnRK1, these transcription factors would either have reduced activities or be degraded. In turn, sugar and amino acid metabolism, oil synthesis, seed maturation and germination, flowering, jasmonic acid, ethylene, and abscisic acid signaling controlled by these transcription factors would be affected	[118]
Sugar sensing and signal trans- duction	*Petunia hybrida*	CaM53	From plasma membrane to nucleus	Loss of geranylation by geranylgeranyl transferases (GGTase-I)	In darkness or at low sugar levels, CaM53 is not geranylated and is localized in the nucleus. With light and sugar accumulation, CaM53 is geranylated by GGTase-I and becomes plasma membrane-bound	[119]

## Data Availability

Not applicable.
